# Single-Cell RNA Sequencing in Multiple Pathologic Types of Renal Cell Carcinoma Revealed Novel Potential Tumor-Specific Markers

**DOI:** 10.3389/fonc.2021.719564

**Published:** 2021-10-14

**Authors:** Cheng Su, Yufang Lv, Wenhao Lu, Zhenyuan Yu, Yu Ye, Bingqian Guo, Deyun Liu, Haibiao Yan, Tianyu Li, Qingyun Zhang, Jiwen Cheng, Zengnan Mo

**Affiliations:** ^1^ Department of Urology, The First Affiliated Hospital of Guangxi Medical University, Nanning, China; ^2^ Institute of Urology and Nephrology, The First Affiliated Hospital of Guangxi Medical University, Nanning, China; ^3^ Center for Genomic and Personalized Medicine, Guangxi Key Laboratory for Genomic and Personalized Medicine, Guangxi Collaborative Innovation Center for Genomic and Personalized Medicine, Guangxi Medical University, Nanning, China; ^4^ Scientific Research Department, The Second Affiliated Hospital of Guangxi Medical University, Nanning, China; ^5^ Department of Urology, Affiliated Tumor Hospital of Guangxi Medical University, Nanning, China

**Keywords:** renal cell carcinoma, single-cell RNA sequencing, ScRNA-seq, tumor-specific markers, endothelial cells, cancer-associated fibroblasts, tumor-immune microenvironment

## Abstract

**Background:**

Renal cell carcinoma (RCC) is the most common type of kidney cancer. Studying the pathogenesis of RCC is particularly important, because it could provide a direct guide for clinical treatment. Given that tumor heterogeneity is probably reflected at the mRNA level, the study of mRNA in RCC may reveal some potential tumor-specific markers, especially single-cell RNA sequencing (scRNA-seq).

**Methods:**

We performed an exploratory study on three pathological types of RCC with a small sample size. This study presented clear-cell RCC (ccRCC), type 2 pRCC, and chRCC in a total of 30,263 high-quality single-cell transcriptome information from three pathological types of RCC. In addition, scRNA-seq was performed on normal kidneys. Tumor characteristics were well identified by the comparison between different pathological types of RCC and normal kidneys at the scRNA level.

**Results:**

Some new tumor-specific markers for different pathologic types of RCC, such as *SPOCK1, PTGIS, REG1A, CP* and *SPAG4* were identified and validated. We also discovered that *NDUFA4L2* both highly expressed in tumor cells of ccRCC and type 2 pRCC. The presence of two different types of endothelial cells in ccRCC and type 2 pRCC was also identified and verified. An endothelial cell in ccRCC may be associated with fibroblasts and significantly expressed fibroblast markers, such as *POSTN* and *COL3A1*. At last, by applying scRNA-seq results, the activation of drug target pathways and sensitivity to drug responses was predicted in different pathological types of RCC.

**Conclusions:**

Taken together, these findings considerably enriched the single-cell transcriptomic information for RCC, thereby providing new insights into the diagnosis and treatment of RCC.

## Introduction

Kidney cancer is a common cancer worldwide; in 2019, it represented 73,820 new cancer cases in the United States (1). Renal cell carcinoma (RCC) is a malignant tumor derived from renal tubular epithelial cells (2). As the most common kidney cancer, RCC is responsible for up to 85% of all cases (3). In addition, the global RCC incidence rates have been increasing in the past decades (4–6). RCC has one of the highest mortality rates in genitourinary cancers, and metastatic RCC (mRCC) occurs in 20% of patients following nephrectomy during follow up (7, 8). RCC is subdivided into several histopathologic and molecular subtypes. Amongst them, clear-cell RCC (ccRCC) has the highest incidence; it accounts for approximately 80% of RCC cases (9). It was followed by papillary RCC (pRCC) and chromophobe RCC (chRCC), which accounted for 10%–15% and 4%–5% of RCC, respectively (9). However, different pathologic types of RCC have different prognosis. In recent years, the application of immune checkpoint inhibitors has been significantly beneficial in metastatic RCC (10, 11). And the magnitude of benefit of the immune checkpoint inhibitors-tyrosine kinase inhibitors combination over sunitinib monotherapy in treatment-naïve metastatic RCC patients is consistent across the clinicopathological subgroups (12). Therefore, studying the pathogenesis of RCC is particularly important and could provide a direct guide for clinical treatment.

*VHL* is the most frequently mutated gene in ccRCC (13, 14). It is mainly mutated through genetic (point mutations or deletions) and/or epigenetic mechanisms leading to the development of cancer (15, 16). Type 1 pRCC is associated with *MET* alterations, whilst type 2 pRCC were characterised by *CDKN2A* silencing, *SETD2* mutations and *TFE3* fusions (17). Although previous studies have provided important clues to the pathogenesis of RCC, they were limited to the DNA level. Given that tumor heterogeneity is probably reflected at the mRNA level, studying mRNA in RCC may reveal tumor heterogeneity, especially single-cell RNA sequencing (scRNA-seq). In a previous study, Kim K et al. (18) first performed scRNA-seq on ccRCC with only more than 100 cells. Although the number of cells was small, a successful demonstration was made for RCC scRNA-seq. Young, M. D et al. (19) used more abundant cells for ccRCC and type 1 pRCC and provided rich transcriptome information. In addition, previous studies have provided in-depth coverage of the tumor immune microenvironment of RCC by using scRNA-seq (20–22). And using scRNA-seq technology, the origin and differentiation of RCC cells are well explained (23, 24). However, there are few reports on the comparison between different pathological types of RCC at single-cell level.

scRNA-seq is a powerful technique for identifying transcriptome characteristic between cells at single-cell resolution. Tumor heterogeneity includes the heterogeneity between different patients and different tumor cells of the same pathological type, which could have prognostic, predictive and therapeutic relevance (2). In this study, we hope to perform an exploratory study on three pathological types of RCC with a small sample size. ScRNA-seq could be used to study the complex cellular features within tumors. The study would also benefit from validating principal findings by deconvoluting bulk RNA-seq data from TCGA and our data, which could help to relate the finding to tumor progression one each subtype. In the present study, the tumor characteristics of RCC can be revealed from the transcriptome level *via* scRNA-seq of the three pathological types of RCC and normal kidney.

## Materials And Methods

### Information of RCC and Normal Kidney Samples

RCC and normal kidney samples (**Table S1**) were obtained from patients undergoing radical nephrectomy at The First Affiliated Hospital and Affiliated Tumor Hospital of Guangxi Medical University (**Figure S1**). The normal tissues were obtained at least 2 cm away from the tumor tissue (**Figure S1**). This study was approved by the Institutional Review Board (IRB) of The First Affiliated Hospital Guangxi Medical University, and all the patients signed the informed consent.

### RCC Sample Procurement and Single-Cell Isolation

Fresh tumor samples were obtained from the operating room to the laboratory in cold Hank’s balanced salt solution (HBSS; Gibco, C11875500BT) with 5% fatal bovine serum (FBS, Gibco, 10099141) and 1% penicillin/streptomycin (P/S; Gibco,15240062). The entire transportation process was within 30 min.

After the samples were washed with 4°C Dulbecco’s phosphate-buffered saline (DPBS; WISENT, 311-425-CL), they were cut into 2–4 mm pieces with sterile scissors. The tissue pieces were washed by resuspending in pre-cold DPBS two times. After the supernatant was removed, the tissue species were digested for 30 min at 37°C with gentle agitation in a digestion solution containing 1 mg/mL of collagenase I (Gibco, 5401020001) and 1 mg/mL of DNaseI (Roche, 10104159001) in HBSS. Then, the digestion was terminated using 10 mL of DMEM (WISENT, 319-006-CL) with 10% FBS (Gibco, 10099141). Subsequently, the suspended cells and tissue fragments went through a 70 μm cell strainer (Falcon), which could filter out large tissue fragments. The cells were washed with pre-cold DPBS containing 300 g of 1% FBS for 5 min two times. Next, red blood cells (RBCs) were removed using 5 mL of RBC lysis buffer (10X diluted to 1X; BioLegend, 420301) for 5 min on ice and then the cells were filtered using a 40 μm cell strainer. Subsequently, they were centrifuged at 300 g for 5 min and washed twice with DPBS. Finally, the cells were resuspended in DPBS with 1% FBS. The single-cell suspension was obtained and viability was calculated using trypan blue (Gibco, 15250-061) staining (**Table S2**). If the cell viability was above 80%, 10x Genomics sample processing was performed.

### Normal Kidney Sample Procurement and Single-Cell Isolation

The preparation for single-cell suspension of three normal kidneys (kidney1, kidney2 and kidney3, **Table S1**) was described in the previous study (25). In this study, the remaining normal kidney tissue (kidney4) was transported in a cold RPMI 1640 (Gibco, C11875500BT) containing 5% FBS and 1% P/S and the entire transport process was completed within 30 min.

This sample was sliced into approximately 2–4 mm pieces and digested for 40 min at 37°C with gentle agitation in a digestion solution containing 0.1 mg/mL of Liberase TL (Roche, 5401020001) and 0.5 mg/mL of DNaseI (Roche, 10104159001) in 5 mL of RPMI 1640. The digestion was then terminated using RPMI 1640 containing 10% FBS. After the suspension was washed with DPBS two times and centrifuged at 300 g for 5 min at 4°C, it was passed through a 70 µm cell strainer. Next, RBCs were removed using 5 mL of 1X RBC lysis buffer for 5 min on ice. Then, the cell suspension passed through a 40 μm cell strainer. After the cells were centrifuged at 300 g for 5 min, they were resuspended in DPBS with 1% FBS. Finally, the single-cell suspension was obtained and live cells were detected *via* trypan blue staining (**Table S2**). If the cell viability was above 80%, 10x Genomics sample processing was performed.

### Sample Processing With 10x Genomics and cDNA Library Preparation

ScRNA-Seq was performed on the above single-cell suspensions in accordance with the standard protocol in the user guide of 10X Genomics Chromium Single Cell 3′ Reagent Kit V3 (https://support.10xgenomics.com/single-cell-gene-expression/index/doc/user-guide-chromium-single-cell-3-reagent-kits-user-guide-v3-chemistry). In brief, the concentration of the single-cell suspensions was manually counted using a haemocytometer and adjusted to 2,000 cells/μL. Appropriate volume was calculated in each sample to catch 10,000 cells. The samples were then loaded into a 10X Genomics single-cell chip. After droplet generation, the samples were transferred onto a PCR tube and reverse transcription reaction was performed using T100 Thermal Cycler (Bio-Rad). Then, cDNA was recovered using a recovery agent provided by 10x Genomics, followed by silane DynaBead clean up as outlined in the Kit V3 user guide. Before the clean-up was performed using SPRIselect beads, the cDNA was amplified for 11–12 cycles.

### ScRNA-Seq Processing and Preliminary Results

All samples were sequenced using Hiseq Xten (Illumina, San Diego, CA) with the following run parameters: read 1 for 150 cycles, read 2 for 150 cycles and index for 14 cycles. Preliminary sequencing files (.bcl) were converted to FASTQ files on CellRanger (version 3.0.2, https://support.10xgenomics.com/single-cell-gene-expression/software/pipelines/latest/what-is-cell-ranger). The 10x Genomics standard protocol was applied to shorten the read 1 end (the barcode and unique molecular identifier) to 26 bp and the read 2 end (mRNA sequence) to 98 bp. The FASTQ files were compared with the human genome reference sequence GRCh38. After CellRanger was used, a barcode table, a feature table and a gene expression matrix were generated.

### Using Seurat for Quality Control (QC) and scRNA-Seq Data Secondary Analysis

R (version 3.5.2, https://www.r-project.org/) and Seurat (26, 27) R package (version 3.1.1, https://satijalab.org/seurat/) were used for QC and secondary analysis. The MergeSeurat function was used to merge the ccRCC samples and the normal kidney samples. Three normal kidney samples (kidney1, kidney2 and kidney3) originated from the previous study (25), whilst the remaining normal tissue (kidney4) was from the same patient of chRCC. Considering the proportion of mitochondrial genes to all genetic material may indicate whether a cell is in homeostasis. For example, type 2 pRCC and ccRCC cells with abundant unique molecular identifiers (UMIs) were mainly found in cells with less than 10% proportion of mitochondrial genes, whilst chRCC cells with abundant UMIs were mainly in less than 30% proportion of mitochondrial genes (**Figures S2A** **–D**). Thus, in accordance with the median number of genes, the percentage of mitochondrial genes and the relationship between the percentage of mitochondrial genes and the mRNA reads (**Figures S2A** **–D**), type 2 pRCC and ccRCC (ccRCC1 and ccRCC2) cells with < 200 and > 5,000 genes (potential cell duplets) and a mitochondrial gene percentage of > 10% were filtered. The chRCC cells with < 200 and > 5,000 genes and a mitochondrial gene percentage of > 30% and the normal kidney cells with < 200 and > 2,500 genes and a mitochondrial gene percentage of > 30% were also filtered (**Figures S2A** **–D**). After filtering was conducted, high-quality RCC cells were obtained and the number of type 2 pRCC, ccRCC, chRCC and normal kidney were 10,132, 12,915, 7,216 and 23,951, respectively. At the same time, given that our data were derived from a small number of samples, we needed to compare the data from scRNA-seq with bulk RNA-seq from TCGA. We selected the differentially expressed genes of pRCC, ccRCC and chRCC from TCGA and integrated into our scRNA-seq data. We found that the results from TCGA were similar to our scRNA-seq data (**Figures S3A** **–C**). In addition, these differentially expressed genes could be precisely mapped to cell types in scRNA-seq data (**Figures S3A** **–C**).

After the data were normalized, all highly variable genes in single cells were identified after controlling for the relationship between average expression and dispersion. All variable genes were used in the downstream analysis, which was the principal-component analysis. R package Harmony (28) (version 0.99.9) was applied to eliminate the batch effect in ccRCC (ccRCC1 and ccRCC2) and the kidney samples (kidney1, kidney2, kidney3 and kidney4). Subsequently, significant principal components (PCs) were identified on the basis of the jackstraw function. Type 2 pRCC, chRCC and the normal kidney used 20 PCs, whilst ccRCC used 25 PCs as the input for uniform manifold approximation and projection (UMAP) when statistically significant. The batch effect between the kidney samples and the ccRCC samples was detected (**Figures S4B** **, C**). With a resolution of 0.25, the cells were clustered using the FindClusters function and classified into nine different cell types in the kidney samples. With a resolution of 0.6, type 2 pRCC and ccRCC were classified into 18 and 21 different cell types, respectively, whilst a resolution of 0.4 was used for chRCC. The FindAllMarkers function was used to find differentially expressed genes (DEGs) between each type of cells (**Tables S3**-**S6**).

### Cell Cycle Analysis

The Seurat program was used for cell cycle analysis. A core set of 43 G1/S and 54 G2/M cell cycle genes were defined on the basis of a previous study (29). Then, the cells were classified by the maximal average expression (‘cycle score’) in these two gene sets. In the case when the cycle scores of G1/S and G2/M were both less than 2, these cells were under non-cycling. Otherwise, they were considered to be proliferative. After cell cycle analysis was performed, no bias induced by cell cycle genes was observed in all samples (**Figures S4D** **–G**).

### Reconstructing Cell Differentiation Trajectories Using Monocle2

The Monocle2 (30) R package (version 2.10.1) was used to reconstruct the cell fate decisions and pseudo-time trajectories of ccRCC cells, chRCC cells, fibroblast and T cells in pRCC. The data of these cells were imported from Seurat object. The genes expressed in at least 10 cells and in greater than 5% of cells were used. Subsequently, the thresholds on the cell local density (rho) and the nearest distance (delta) were used to determine the number of clusters. Then, differential gene expression analysis was conducted across all cell clusters. The top 1000 most significant DEGs were used for the set of ordering genes and dimension reduction and trajectory analysis were performed. Once a trajectory was established, these key genes that varied with pseudo-time could be discovered using the differential GeneTest function.

### Comparing Present scRNA-Seq Data With Those of Previous Studies

The scRNA-seq data of three normal kidneys (kidney1, kidney2 and kidney3) came from a previous study, GSE131685 (25). Other normal kidney data were obtained from a previous study (31) and available through the Human Cell Atlas data portal (https://data.humancellatlas.org/explore/projects/abe1a013-af7a-45ed-8c26-f3793c24a1f4). UMAP plot representation of 23,951 normal kidney cells from four different samples (**Figures S4A**).

### Integration of scRNA-Seq Results With Genome-Wide Association Study (GWAS) and The Cancer Genome Atlas (TCGA) Databases

Here, the methods used were based from a previous study (32). All the GWAS genes associated with RCC were downloaded from the GWAS catalogue (33) (downloaded 10 February 2020). Using renal cell carcinoma as keywords, the GWAS catalogue was searched and the data were downloaded. The genes with *p* value greater than 5 × 10^−8^ were filtered out and obtained for subsequent correspondence with cell types by using scRNA-seq (**Table S7**).

Some special genes were discovered using scRNA-seq; they may be associated with the prognosis of RCC. Then, these genes were integrated into the TCGA dataset (17) and the GEPIA (34) tool was used to plot the Kaplan–Meier survival curves. The patients were divided into high-risk and low-risk groups, with a cut-off value of 50%, and the hazards ratio (HR) used 95% CI. The top 60 DEGs in chRCC1 and chRCC3 were selected. A total of 25 DEGs were found in chRCC2. The *p* value less than 0.05 was used to predict prognostic genes.

### Gene Ontology (GO) Enrichment Analysis on Different Types of Tumor Cells

In accordance with the DEGs calculated using Seurat, the top 50 DEGs in each tumor cell type (type 2 pRCC, ccRCC and chRCC) were selected for GO enrichment analysis (35) (http://geneontology.org/). Only 25 DEGs were found in chRCC 2 and then all the DEGs were selected for GO enrichment analysis. Each tumor cell type underwent enrichment analysis of biological process and the 15 most significant biological processes were shown (**Table S8**).

### Ligand–Receptor Interactions

The ligand–receptor interaction score was calculated with reference to a previous study (36). In brief, the ligand–receptor interaction scores between three different types of RCC cells and cancer-associated fibroblasts (CAFs), together with immune cells, were calculated. The higher ligand–receptor interaction score reflected the stronger potential interaction between the cells. The ligand–receptor pairs with scores greater than 1 were listed.

### Prediction of Activation of Drug Target Pathways and Sensitivity to Drug Responses

The GSVA algorithm (37) was used to evaluate the relative activation status of pathways in different pathological types of RCC in scRNA-seq data. In the previous studies (2, 38–40), the progression of RCC may be associated with the activation of many signaling pathways. Twelve targeted pathways were selected: EGFR pathway, FGFR pathway, MAPK pathway, MET pathway, mTOR pathway, PDGFRA pathway, PDGFRB pathway, PI3K/AKT pathway, RAF pathway, SCF-KIT pathway, SRC pathway and VEGFR pathway. The GSVA scores were transformed to binary scores to evaluate whether these gene signatures were significantly activated. The gene sets with same size and each original panel of genes were randomly generated with permutation (×1000) and then calculated for the GSVA scores. The original GSVA scores were defined as ‘activated’ by the cut-off values of the averaged scores in the randomly selected gene sets.

The related targeting drug sensitivities were also predicted in different pathological types of RCC. In accordance with a previous study (16), the Cancer Genome Project (41), which includes measured drug response data from cancer cell line expression data, was used as a training set. Leave-one-out cross validation (18) was applied to analyze the total dataset and evaluate the prediction sensitivity. A total of 13 common targeted drugs (afatinib, axitinib, cabozantinib, crizotinib, dasatinib, erlotinib, foretinib, gefitinib, pazopanib, selumetinib, sorafenib, sunitinib and temsirolimus) were used to predict drug sensitivity. The results were transformed into Z-scores. The nanomolar-scaled IC50 values were also transformed into Z-scores to ensure accurate prediction of drug sensitivity.

### Immunohistochemistry

Each immunohistochemistry paraffin (IHC-P) result was verified in five patient samples (**Table S10**). Each antibody was performed in at least three slides. RCC and normal kidney tissues were obtained from the Department of Pathology at The First Affiliated Hospital of Guangxi Medical University. The tissue slices were rehydrated using solutions of ethanol ranging from 100% to 70% and washed with PBS (Solarbio; P1010-2). After the slides underwent high-pressure repair using sodium citrate (Solarbio; C1032), the tissues in goat serum (ZSGB-BIO; SP-9000) were blocked with PBS for 15 min at room temperature. Subsequently, the slides were incubated with anti-*SPOCK1* (rabbit anti-human/mouse, 1:100, Abcam; ab229935), anti-*PTGIS* (rabbit anti-human/mouse/rat, 1:100, Abcam; ab23668), anti-*NDUFA4L2* (rabbit anti-human, 1:100, Abcam; ab190007), anti-*REG1A* (rabbit anti-human, 1:100, Abcam; ab47099), anti-*RHCG* (rabbit anti-human, 1:500, Novus; NBP2-30905) and anti-*SPAG4* (rabbit anti-human, 1:50, Novus; NBP2-38937) antibodies and PBS control group prepared in blocking solution at 4°C overnight. The tissues then were incubated with secondary antibody (ZSGB-BIO; SP-9001) for 15 min and with tertiary antibody (ZSGB-BIO; SP-9001) for 15 min at room temperature after washing by PBS. Finally, the slides were stained with DAB and nucleated with haematoxylin.

The same method was used to perform IHC-P in human normal kidney tissues for the control groups. Anti-*SPOCK1*, anti-*PTGIS*, anti-*NDUFA4L2*, anti-*REG1A*, anti-*RHCG* and anti-*SPAG4* antibodies were also used (**Figures S5A** **–F**).

### Immunofluorescence (IF)

Before IF was performed, the antibody specificity was confirmed by labelling the control groups. Two negative controls were set: PBS and anti-mouse secondary antibody (Alexa Fluor 488, goat anti-mouse IgG pre-adsorbed, 1:500, Abcam; ab150117) and PBS and anti-rabbit secondary antibody (Alexa Fluor 594, goat anti-rabbit IgG pre-adsorbed, 1:500, Abcam; ab150084). These results indicated no unspecific reaction occurred in secondary antibodies (**Figure S5G**). The same protocol as IHC-P was used until high-pressure repair and incubation with primary antibodies were finished. Then, the following groups were set to verify the results: *ACTA2* (mouse anti-rabbit/rat/human, 1:200, Abcam; ab7817) and *KRT8* (rabbit anti-mouse/human, 1:200, Abcam; ab53280), *KI67* (rabbit anti-mouse/rat/human, 1:1,000, Abcam; ab15580) and *PDGFRB* (mouse anti-rat/human, 1:200, Abcam; ab69506), *KI67* and *CD68* (mouse anti-human/rat/rabbit, 1:100, Abcam; ab955), *KI67* and CD3 (mouse anti-human, 1:50, Abcam; ab699), *CD31* (mouse anti-human, 1:1, 000, Abcam; ab9498) and *POSTN* (rabbit anti-mouse/rat/human, 1:100, Abcam; ab14041) and *CD31* (rabbit anti-mouse/human, 1:300, Abcam; ab28364) and *COL3A1* (mouse anti-rat/human, 1:100, Abcam; ab6310). After the slides were incubated with the primary antibodies at 4°C overnight, they were incubated with secondary antibodies Alexa Fluor 488 and Alexa Fluor 594 at 37°C for an hour. Finally, the slides were stained with DAPI (Abcam, ab104139) for 10 min.

### Western Blot

The Western blot results were verified in five patient samples (**Table S10**) and repeated at least twice. The human normal kidney (100mg) and ccRCC (106mg) tissues were lysed with RIPA lysis buffer containing both protease Inhibitor and phosphatase inhibitor on ice. We collected the supernatant and used a BSA Quantification Kit to determine the protein concentrations after centrifugation at 12,000 rpm for 10 minutes. Protein samples (40mg) from supernatants were separated on SDS-PAGE and transferred onto polyvinylidene difluoride membrane. The membrane was blocked for 1 hour with blocking buffer containing 5% nonfat milk. After three times washings with TBST, membranes were incubated at 4°C overnight with primary antibodies, anti-Ceruloplasmin (rabbit anti-human, 1:1000, abcam, ab48614) and anti-GAPDH (mouse anti-human/mouse/rat, 1:5000, abcam, ab8245). The membranes were washed three times with TBST and incubated with secondary antibodies at room temperature for 1 hour, and then washed three times again. Immunoreactivity was visualized by an imager (ImageQuant LAS 500; GE Healthcare). GAPDH was used for a loading control. The presented results are from at least three repetitions of Western blot. Except the primary antibody and GAPDH were Abcam, the other reagents were used the western blotting kit from BOSTER Biological Technology co. Itd (AR0040).

### Cell-Type Markers

The RCC cell types were defined in accordance with the marker genes reported in previous studies (13, 17, 42–63) (**Table 1**). The cell type of normal kidney was assigned on the basis of the previous study (25).

**Table 1:** Cell-type assignment based on the marker genes reported in previous studies.

Cell type	Markers	Cell type	Markers
pRCC	*CDKN2A* (17)	ccRCC	*CA9* (13)*, NDUFA4L2* (49)
chRCC	*RHCG* (42)	CD8+ T cells	*CD3D* (43, 44, 58)*, CD3E* (43, 44, 58)*, CD8A* (43, 44, 58)
Macrophage	*CD68* (58)*, CD163* (58),	CD4+ T cells	*CD3E* (43, 44, 58)*, CD3D* (43, 44, 58)*, IL7R* (44, 63)
Monocyte	*CD14* (46, 48, 57)*, LYZ* (57, 58)*, S100A12* (58)*, S100A9* (58)*, S100A8* (58)	B cells	*CD79A* (43, 57)*, CD79B* (43, 57)*, MS4A1* (57)
Dendritic cells	*FCER1A* (46–48)*, CD1E* (46–48)*, CD1C* (46–48)*, HLA-DMA* (46–48)*, HLA-DMB* (46–48)	Plasma cells	*IGKC* (45)
NK cells	*KLRD1* (61)*, KLRC1* (61)	Mast cells	*TPSAB1* (62)*, TPSB2* (62)*, KIT* (59)
Fibroblast	*SFRP2* (51)*, SPARC* (60)*, MMP2* (52, 53)*, COL3A1* (55)*, COL1A1* (55, 56)*, COL1A2* (55, 56)*, EMILIN1* (54)*, PDGFRB* (52)	CAF	*ACTA2* (52)*, TAGLN* (53)
Endothelial cells	*PECAM1* (50)*, PLVAP* (50)*, CDH5* (50)*, KDR* (50)	TAM	*GPNMB* (58)*, SLC40A1* (58), MSR1 (64)

## Results

### Single-Cell Transcriptomic Atlas of Multiple Pathologic Types of RCC and Normal Kidney

**Figure 1:**
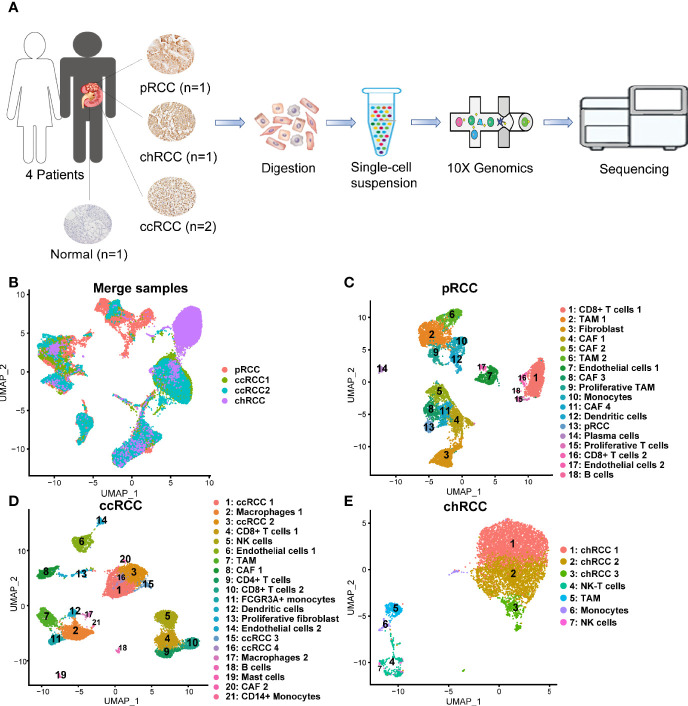
Single-cell transcriptomic atlas of multiple pathologic types of RCC and human kidney. **(A)** Schematic of the overall study design. ScRNA-seq was applied to type 2 pRCC, ccRCC, chRCC and the normal kidney on 10x Genomics Chromium platform. **(B)** UMAP plot representation of 30,263 tumor cells from three pathological types of RCC. **(C)** Uniform manifold approximation and projection (UMAP) plot representation of 10,132 type 2 pRCC cells with 18 distinct cell types. **(D)** UMAP plot representation of 12,915 ccRCC cells from two different samples with 21 distinct cell types. **(E)** UMAP plot representation of 7,216 chRCC cells with seven distinct cell types.

ScRNA-seq was performed in seven different patients, including four tumor samples and four normal kidney samples (kidney1, kidney2 and kidney3 came from our previous study (25), and kidney4 from this study) to explore the cellular diversity and gene expression characteristics in RCC (**Figure 1A** and **Table S1**). After QC was conducted using Seurat (26, 27), ccRCC, type 2 pRCC and chRCC were presented in 30,263 high-quality single-cell transcriptome information. We performed merge UMAP of four tumor samples (**Figure 1B**). Meanwhile, scRNA-seq was performed on one normal kidney, providing a total of 585 single-cell transcriptome information.

Single-cell transcriptomes were acquired in a total of 10,132 cells from type 2 pRCC. They could be classified into 18 different cell types (**Figure 1C**). On the basis of the marker genes (**Figure S6A** and **Table 1**), these cell types were defined from cluster 1 to 18 as CD8+ T cells 1, tumor-associated macrophage (TAM) 1, fibroblast, CAF 1, CAF 2, TAM 2, endothelial cells 1, CAF 3, proliferative TAM, monocytes, CAF 4, dendritic cells, pRCC, plasma cells, proliferative T cells, CD8+ T cells 2, endothelial cells 2 and B cells (**Figure 1C**). In ccRCC, a total of 12,915 single-cell transcriptome information were acquired from two samples, including 21 different cell types corresponding to ccRCC 1, macrophages 1, ccRCC 2, CD8^+^ T cells 1, NK cells, endothelial cells 1, TAM, CAF 1, CD4^+^ T cells, CD8^+^ T cells 2, FCGR3A^+^ monocyte, dendritic cells, proliferative fibroblast, endothelial cells 2, ccRCC 3, ccRCC 4, macrophages 2, B cells, mast cells, CAF 2 and CD14^+^ monocytes (**Figure 1D**) by the marker genes (**Figure S6B** and **Table 1**). In chRCC, 7,216 high-quality cells were further analysed and clustering analysis identified seven distinct cell clusters. In accordance with the marker genes (**Figure S6C** and **Table 1**), these cells could be classified as chRCC 1, chRCC 2, chRCC 3, NK-T cells, TAM, monocytes and NK cells (**Figure 1E**).

In addition, 23,951 normal kidney cells were further analysed and nine distinct cell clusters were identified. In accordance with the marker genes (**Figure S6D**), the cells were classified into clusters 1–9 corresponding to proximal convoluted tubule cells, proximal tubule cells, glomerular parietal endothelial cells, proximal straight tubule cells, NK-T cells, monocytes, distal tubule cells, collecting duct (CD) cells and B cells.

### Diversity and Gene Expression Characteristics in RCC Cells as Revealed by scRNA-Seq

**Figure 2:**
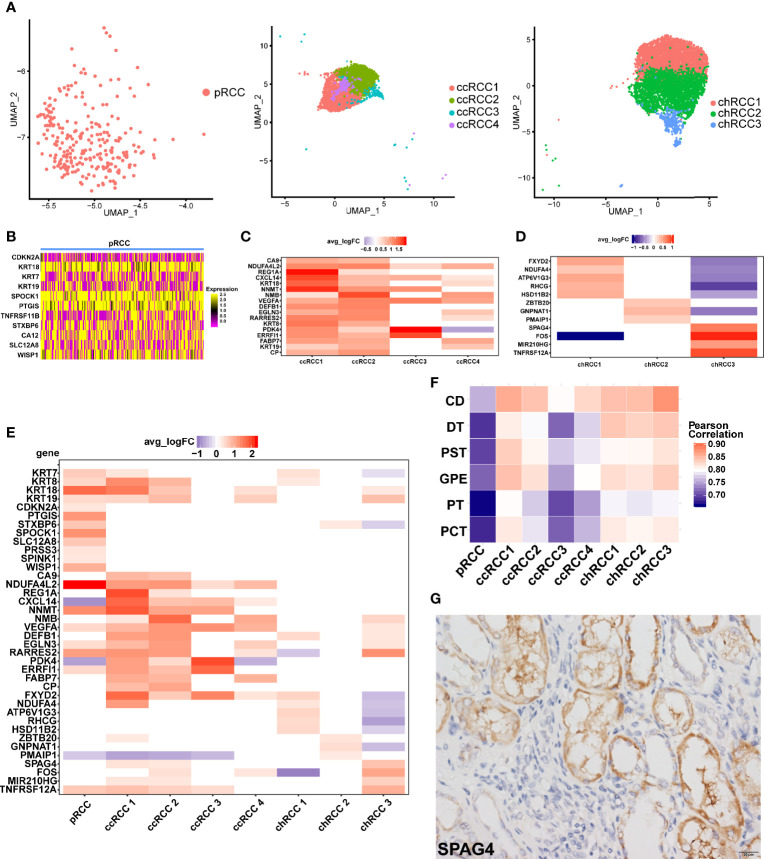
ScRNA-seq revealed tumor heterogeneity of RCC. **(A)** UMAP plot of the tumor cells in different pathologic types (type 2 pRCC, ccRCC and chRCC). **(B–D)** DEGs of different pathologic types of RCC, as shown by scRNA-seq, avg logFC and average log2 fold change. **(E)** Comparison of DEGs in different pathological RCCs. **(F)** Comparison of normal kidney cell types with RCC cell types by calculating the Pearson correlation coefficient. CD, collecting duct cells; DT, distal tubule cells; PST, proximal straight tubule cells; GPE, glomerular parietal epithelial; PT, proximal tubule cells; PCT, proximal convoluted tubule cells. **(G)** IHC-P verification of *SPAG4* positive cells in chRCC tissue. Scale bars, 20 μm.

Unlike bulk RNA sequencing, scRNA-seq could study the transcriptome of tumor cells at single-cell resolution. In this study, unbiased clustering analysis not only precisely defined type 2 pRCC but also classified ccRCC and chRCC into several different tumor cell types (**Figure 2A**). Except for the marker gene *CDKN2A* (17), epithelial-derived *KRT18* and *KRT7*, type 2 pRCC also differentially expressed *SPOCK1*, *PTGIS* and other genes (**Figure 2B**). In ccRCC, obvious differences in gene expression were found amongst four ccRCC cell types (**Figure 2C**). For instance, ccRCC1 and ccRCC2 highly expressed the ccRCC markers *CA9 (* 13 *)* and *NDUFA4L2* (49), whilst ccRCC3 and ccRCC4 only expressed *NDUFA4L2* (**Figure 2C**). The DEGs amongst three chRCC cell types were also found (**Figure 2D**). The DEGs amongst the tumor cells of three pathological RCCs were compared (**Figure 2E**).

On the basis of the DEGs, GO enrichment analysis was performed on each tumor cell type and the results showed that the biological process (BP) of type 2 pRCC was mainly concentrated in ‘cell adhesion’ and ‘biological adhesion’ (**Figure S7A**). CcRCC1 was concentrated in ‘response to decreased oxygen levels’, ‘response to oxygen levels’ and ‘response to hypoxia’ (**Figure S7B**). Considering that the pathogenesis of ccRCC was related to hypoxia caused by *VHL* mutation (65), ccRCC1 may be associated with this process. GO enrichment analysis was also conducted on the other tumor cell types (**Figures S7C** **–H**).

The correlation of average gene expression between tumor cells and normal cells was compared to explore the origin of tumor cells. We found that chRCC3 was highly correlated with CD cells (**Figure 2F**). Considering the specificity of chRCC3 with high expression of *SPAG4* (**Figure 2D**), this marker was verified in the chRCC tissue *via* IHC-P. The positive cells were clustered around the CD cells (**Figure 2G**), which further supported the hypothesis.

### Identification and Verification of Some Novel Tumor-Specific Gene Markers

**Figure 3:**
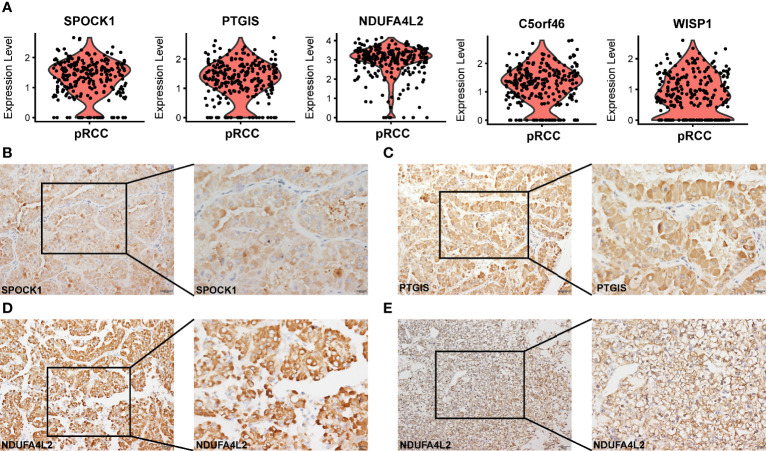
Tumor-specific markers in different types of pRCC. **(A)** Violin plots showing tumor-specific markers expressed in type 2 pRCC. **(B–D)** IHC-P verification of tumor-specific markers (*SPOCK1, PTGIS* and *NDUFA4L2*) in type 2 pRCC (**Table S10**). Scale bars, 20 μm (left) and 50 μm (right). **(E)** IHC-P verification of tumor-specific marker (*NDUFA4L2*) in ccRCC. Scale bars, 20 μm (left) and 50 μm (right).

An important advantage of scRNA-seq is its ability to classify cells precisely and discover the characteristics of gene expression. For tumor cells, the specific genes that were expressed significantly were identified. In type 2 pRCC, five significant candidate genes were identified (*SPOCK1*, *PTGIS*, *NDUFA4L2*, *C5orf46* and *WISP1*); they were generally highly expressed in type 2 pRCC (**Figure 3A**). Subsequently, to confirm that these genes were tumor-specific, their expression was enriched in the scRNA-seq data from normal kidneys. The present study and the data in a Science paper (31) demonstrated very low or no expression of these five genes (**Figures S8A, B**). In addition, three tumor-specific genes, namely, *SPOCK1*, *PTGIS* and *NDUFA4L2*, were verified using IHC-P in type 2 pRCC tissues (**Figures 3B** **–D**) and compared with the negative controls in normal kidney tissues (**Figures S5A** **–C**). Interestingly, in a previous study *NDUFA4L2* was a marker for ccRCC (49). However, we discovered that this gene was also highly expressed in type 2 pRCC (**Figures 3D, E**).

**Figure 4:**
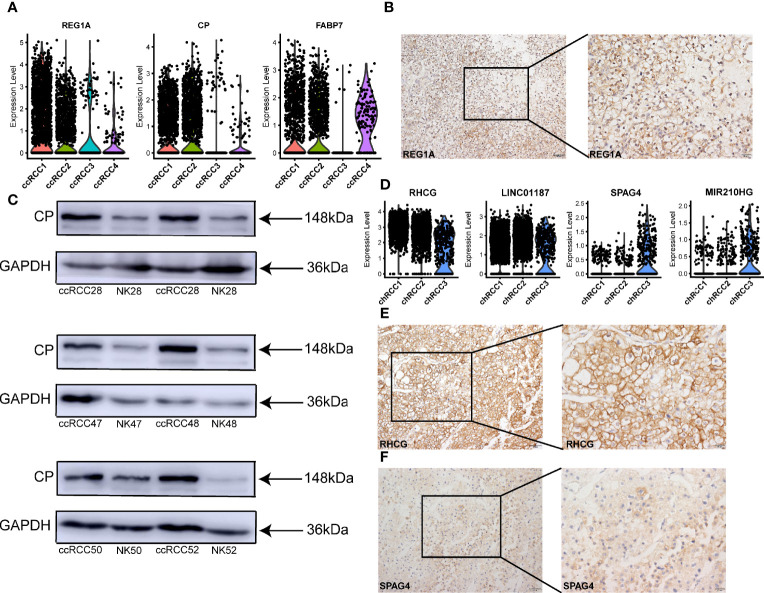
Tumor-specific markers in different types of ccRCC and chRCC. **(A)** Violin plots showing tumor-specific markers expressed in ccRCC. **(B)** IHC-P verification of tumor-specific marker *REG1A* in ccRCC. Scale bars, 20 μm (left) and 50 μm (right). **(C)** Western blot was performed showing the expression of CP protein in different ccRCC tissues (**Table S10**) and normal kidney (NK). **(D)** Violin plots showing tumor-specific markers expressed in chRCC. **(E, F)** IHC-P verification of tumor-specific markers *RHCG* and *SPAG4* in chRCC (**Table S10**). Scale bars, 20 μm (left) and 50 μm (right).

Three specific candidate genes (*REG1A*, *CP* and *FABP7*) were also identified in ccRCC (**Figure 4A**) and compared with those in the normal kidney (**Figures S8C, D**). Then, the expression of *REG1A* in ccRCC tissues was verified using IHC-P but not in normal kidney tissues (**Figures 4B**, **S4D**). And the expression of *CP* in ccRCC tissues was higher than that in normal kidney tissues (**Figure 4C**). In a previous study, *RHCG* and *LINC01187* are identified as marker genes for chRCC (42). In the present study, we also verified the previous results (**Figures 4D, E**, **S5E**, **S8E, F**), which further enhanced the reliability of our chRCC data and tumor cells’ definition. In addition, a novel tumor-specific gene marker called *SPAG4* was discovered in chRCC and it was more specifically expressed in chRCC3 (**Figure 4D**). A positive result in chRCC tissues was obtained *via* IHC-P (**Figure 4F**), whilst a negative result was obtained in normal kidney tissue (**Figure S5F**). Thus, the results identified some new tumor-specific markers and verified *SPOCK1*, *PTGIS*, *REG1A, CP* and *SPAG4* in different types of RCC. And *NDUFA4L2* both highly expressed in tumor cells of ccRCC and type 2 pRCC.

### Phylogenetics and Evolution of RCC as Revealed by scRNA-Seq

**Figure 5:**
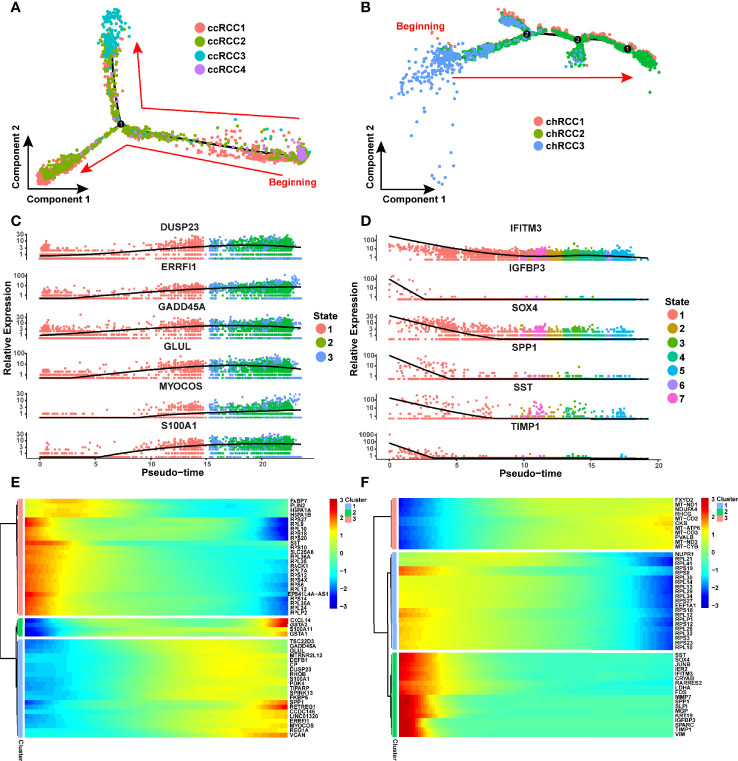
CcRCC and chRCC development trajectories reconstructed using Monocle2. **(A, B)** Pseudo-time trajectories performed on ccRCC and chRCC tumor cells. Each dot stands for one cell and is colored in accordance to its cell type. The arrows represent the direction of cell evolution. **(C)** Scatter plots showing the expression levels and changes in relative expression of key genes that affected the evolution of ccRCC with pseudo-time. **(D)** Scatter plots showing the expression levels and changes in relative expression of key genes that affected the evolution of chRCC with pseudo-time. **(E, F)** Heatmap showing the top 50 genes that affected the evolution of ccRCC and chRCC cells along the trajectory.

The evolution of tumor cells has always been a hot topic in oncology. Although previous study has predicted the putative cell of origin for more than 10 RCC subtypes by using a random forestmodel trained (24), we hope to apply Monocle2 (30) to reconstruction the different tumor cell subtypes differentiation trajectory of ccRCC and chRCC. In this study, Monocle2 was used to construct the evolutionary trajectory of these cancer cells on the basis of the single-cell transcriptome information. The developmental trajectory of ccRCC was reconstructed. CcRCC4 was almost at the beginning stage of development, whilst ccRCC3 was almost at the end stage of the development trajectory. CcRCC1 and ccRCC2 were present throughout the trajectory (**Figure 5A**). The top six genes that were most critical to the development of ccRCC were identified as *DUSP23*, *ERRFI1*, *GADD45A*, *GLUL*, *MYOCOS* and *S100A1* (**Figure 5C**). In chRCC, 6,437 tumor cells were included for analysis using the same method. ChRCC3, which specifically expressed *SPAG4*, was at the beginning of the trajectory, whilst chRCC1 and chRCC2 were present throughout the development trajectory (**Figure 5B**). The top 6 key genes that influenced the development trajectory was also highlighted. They were *IFITM3*, *IGFBP3*, *SOX4*, *SPP1*, *SST* and *TIMP1* (**Figure 5D**). These genes were divided into three clusters *via* a pseudo-temporal expression pattern to further explore the genes that changed in pseudo-time. In ccRCC and chRCC, the top 50 genes, which varied as a function of pseudo-time, were clustered, as shown by the heat map (**Figures 5E, F**).

### Diversity of Fibroblasts (Including Multiple CAFs) in Type 2 pRCC as Revealed by scRNA-Seq

**Figure 6:**
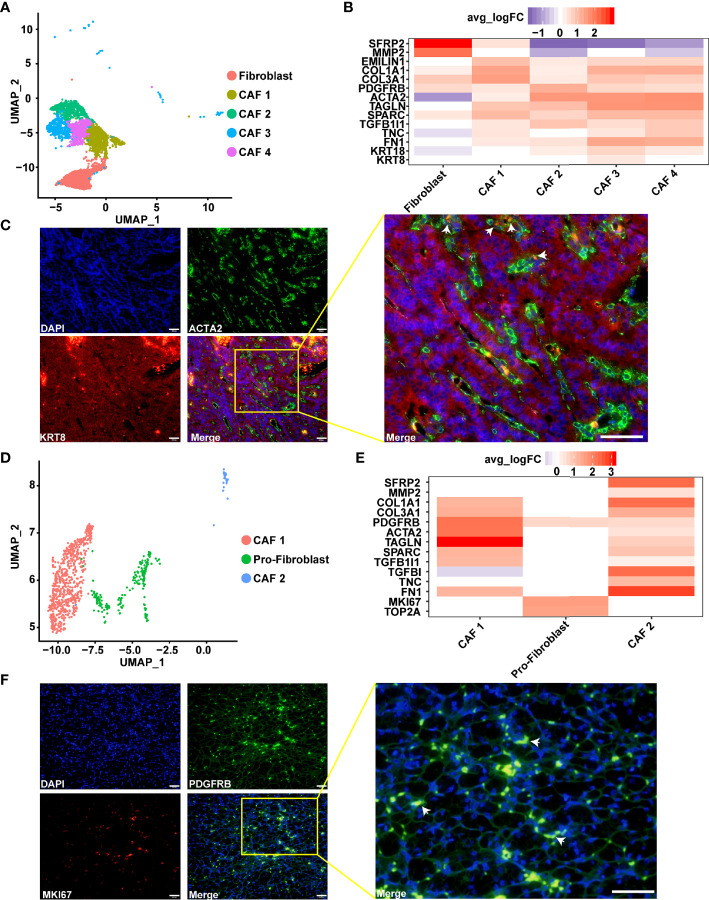
Diversity of CAFs as identified using scRNA-seq. **(A)** UMAP plot showing the subpopulation of fibroblast in type 2 pRCC. **(B)** Gene expression characteristics of the fibroblasts in type 2 pRCC, including four types of CAF. **(C)** IF analysis of the expression of *ACTA2* (green), which is a CAF marker, in combination with the epithelial cell marker *KRT8* (red) and DNA staining using DAPI (blue) within the paraffin sections from human type 2 pRCC samples. CAF 3 (arrows) was verified using IF. Scale bars, 50 μm. **(D)** UMAP plot showing the subpopulation of fibroblast in ccRCC. **(E)** Gene expression characteristics of the fibroblasts in ccRCC, including two types of CAF and proliferative fibroblast (pro-fibroblast). **(F)** IF analysis of the expression of *PDGFRB* (green), which is a fibroblast marker, in combination with the marker *MKI67* (red) and DNA staining using DAPI (blue) within the paraffin sections to verify the pro-fibroblast (arrows). Scale bars, 50 μm.

Fibroblasts, especially CAF, are major components of the tumor microenvironment and play an important role in tumor progression (66). Previous studies have described the diversity of CAFs in breast cancer *via* fluorescence-activated cell sorting (67). In the present study, multiple CAFs were discovered in type 2 pRCC and ccRCC through scRNA-seq (**Figures 6A, D**). In type 2 pRCC, these cells could be classified into four CAF cell types and one quiescent fibroblast. The quiescent fibroblast highly expressed the markers of fibroblast, namely, *SFRP2* (51) and *MMP2* (52, 53), but did not express the markers of CAF, namely, *ACTA2* (52) and *TAGLN* (53) (**Figure 6B**). All the CAFs in type 2 pRCC expressed *TGFB1I1* (**Figure 6B**), which reflected the exocrine phenotype of CAFs (68). CAF 2, CAF 3 and CAF 4 expressed these markers associated with epithelium, especially CAF 3, which expressed *KRT8* and *KRT18* (**Figure 6B**). These CAFs may be epithelial-to-mesenchymal transition (EMT) and retain epithelial characteristics. Thus, the spatial location of CAF 3 in type 2 pRCC tissues was validated using IF (**Figure 6C**). CAF 3 was very close to the tumor cells.

Two subpopulations of CAFs, which expressed *ACTA2* (52) and *TAGLN* (53), the markers of CAF, were discovered in ccRCC (**Figure 6E**). CAF 1 highly expressed *ACTA2* and *TAGLN*, whilst CAF 2 specifically expressed *SFRP2*, *MMP2*, *TGFBI* and *TNC*. *TGFBI* encodes transforming growth factor-β, which is an important secretion of CAF (66, 68). Thus, CAF 2 was a secretory phenotype of CAF in ccRCC. The fibroblast that specifically expressed the proliferation factor *MKI67* in ccRCC was discovered. In view of a previous report of proliferative T cells in hepatocellular carcinoma by scRNA-seq (58), these cells were named proliferative fibroblast. In addition, this result was verified in ccRCC (**Figure 6F**).

We found that the fibroblast subpopulations of pRCC were more abundant than that of ccRCC. In pRCC, the CAFs would be associated with the EMT process, while this characteristic was not found in ccRCC. Based on the spatial localization of CAFs and tumor cells, it was inferred that this could be caused by the interaction between tumor cells and fibroblasts (**Figure 6C**). In addition, the expression of CAFs’ marker genes in ccRCC and pRCC were very similar, except proliferative fibroblast (**Figures 6B** **, E**).

### Two Types of Endothelial Cells in Type 2 pRCC and ccRCC and a Type of Endothelial Cells Associated With Fibroblast in ccRCC

**Figure 7:**
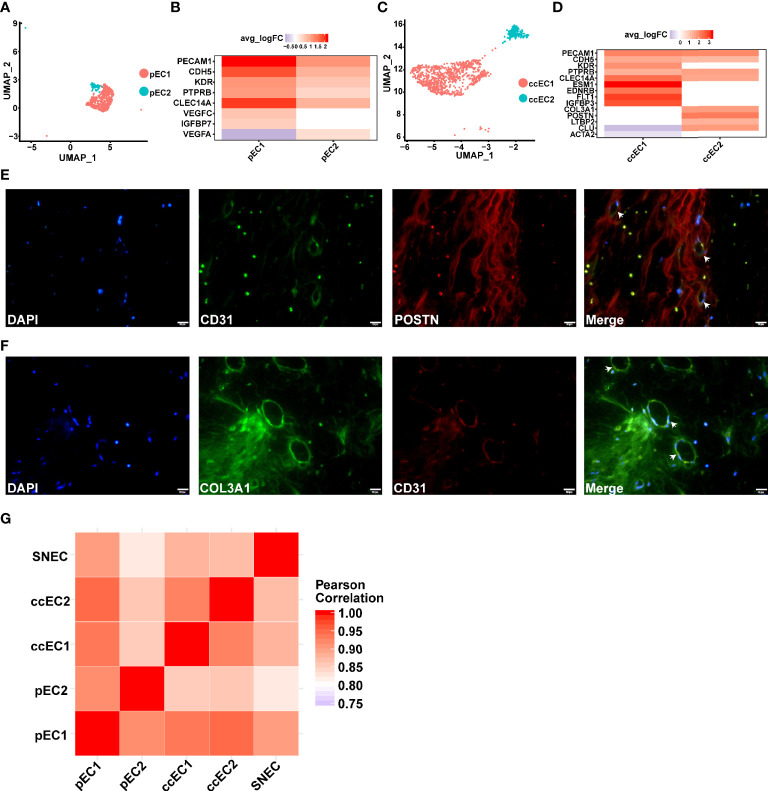
scRNA-seq revealed two subpopulations of ECs in pRCC and ccRCC. (A–D) Two types of endothelial cells discovered in type 2 pRCC and ccRCC, respectively. **(A)** UMAP plot representation of two types of ECs in type 2 pRCC. **(B)** DEGs of two types of ECs in type 2 pRCC. **(C)** UMAP plot representation of two types of ECs in ccRCC. **(D)** DEGs of two types of ECs in ccRCC. A novel EC type (POSTN+ and COL3A1+) was identified. **(E)** IF analysis of the expression of CD31 (green), which is an EC marker, in combination with *POSTN* (red) and DNA staining using DAPI (blue) within the tissue paraffin sections from ccRCC to verify these novel ECs. **(F)** Another marker, *COL3A1* (green), used to verify the same result. Scale bars, 20 mm. **(G)** Heat map indicating Pearson correlation coefficient on the average gene expression amongst EC population in type 2 pRCC, ccRCC and normal kidney.

Two types of endothelial cells (ECs) were found in type 2 pRCC and ccRCC through scRNA-seq (**Figures 7A, C**). In type 2 pRCC, EC could be classified into two cell subtypes, namely, pEC1 and pEC2 (**Figure 7A**). Although both types of EC expressed classical endothelial cell markers, such as *PECAM1 (* 50), *CDH5 (* 50) and *KDR (* 50) (**Figure 7B**), a significant heterogeneity was observed between them. The heterogeneity between two types of EC was mainly reflected in the expression of endothelial growth factor (*VEGF*). The endothelial cell 1 of type 2 pRCC (pEC1) specifically expressed *VEGFC*, whilst pEC2 significantly expressed *VEGFA* (**Figure 7B**). Considering that *VEGF* is closely related to tumor progression and prognosis, accurate classification of EC and in-depth understanding of their potential biological functions may be very helpful for the treatment of RCC.

A type of EC with fibroblast characteristics was identified in ccRCC. It expressed *COL3A1* (55) and *POSTN* (**Figure 7D**). Although the endothelial-to-mesenchymal transition to CAF has been reported in previous studies (68), these cells did not express the markers of CAF (**Figure 7D**). Given that fibroblast-like EC has not been previously reported, it may only exist in specific tumor tissues, such as ccRCC. Therefore, these cells expressing *PECAM1* and *POSTN* or *COL3A1* in ccRCC tissues were labelled. The results indicated that this type of EC expressing fibroblast markers was indeed present in ccRCC (**Figures 7E** **, F**). Then, the gene expression similarities between tumor EC and normal renal EC were compared [data from a previous study (31)]. The result of Pearson correlation coefficient demonstrated that the average gene expression in these endothelial cells was very similar (**Figure 7G**). The heterogeneity amongst these cells may be due to the differences in the expression of a few genes.

### Discovery of Some Characteristics in Tumor-Immune Microenvironment *via* Comparison With Normal Kidney

**Figure 8:**
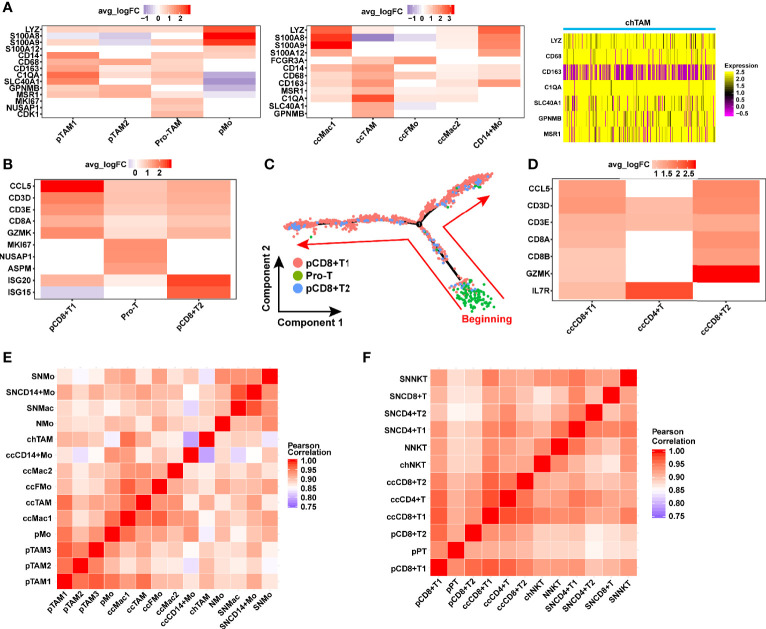
Identification of the macrophage and T cell in TME of RCC. **(A)** Gene markers distinguishing the various types of macrophage in different TMEs of RCC. **(B, D)** Heat map showing gene expression of T cell in pRCC and ccRCC. **(C)** Monocle2-generated pseudo-temporal trajectory of three types of T cell. The arrows represent the direction of cell evolution. **(E, F)** Comparison of the correlation of TAM and T cells between tumor and normal kidney. (SN: Science paper’s normal kidney, Mo, monocytes; Mac, macrophage; FMo, FCGR3A^+^ monocyte; N, normal kidney in this study; ch, chRCC; cc, ccRCC; p, pRCC; TAM, tumor-associated macrophage; NKT, natural killer T cell; pPT, pro-T cells in pRCC).

Monocytes/macrophages were involved in three pathologic RCCs (**Figures 1C** **–E**). Especially in pRCC and ccRCC, monocytes/macrophages were classified into many subpopulations by gene markers (**Figure 8A**). TAM was present in three pathologic RCC, which was defined by *GPNMB* (58), *SLC40A1* (58) and *MSR1* (64). Three types of TAM in pRCC were found, namely, proliferative TAM (Pro-TAM), TAM 1 and TAM 2. Pro-TAM not only expressed TAM markers but also specifically expressed the proliferation factor *MKI67* (**Figure 8A**). Although Pro-TAM was previously reported in pRCC (69), the transcriptomic characteristics of such cell in type 2 pRCC have not been reported. In addition, the Pro-TAM was not found in ccRCC or chRCC (**Figure 8A**), which may be characteristic of the tumor immune microenvironment in pRCC.

T cells are immune cells with tumor-killing characteristic, especially CD8^+^ T cells, which were involved in pRCC and ccRCC (**Figures 1C** **, D**). In ccRCC, T cells included CD8^+^ T cells 1, CD8^+^ T cells 2 and CD4^+^ T cells (**Figure 8D**). In pRCC, T cells could be classified into three cell types, namely, CD8^+^ T cells 1, CD8^+^ T cells 2 and proliferative T cells (**Figure 1C**). In accordance with the characteristics of gene expression, a type of T cells specifically expressing *MKI67* was found and regarded as proliferative T cells (**Figure 8B**). Here we found that proliferation T cells infiltrating was a feature of the ccRCC immune microenvironment which was consistent with previously studies (20–22, 70). Pseudo-time trajectory analysis on all T cells was performed in pRCC to further understand the proliferation characteristics of this cell type. The result also verified the feature of proliferative T cells that were almost at the beginning stage of development trajectory (**Figure 8C**).

Considering the important role of monocytes/macrophages in tumor microenvironment, the correlation of monocyte/macrophage gene expression between three pathologic types of RCC and normal kidney tissue was compared. The monocytes/macrophages in RCC had a very high correlation with their gene expression (**Figure 8E**). However, the correlation between the gene expression of monocytes/macrophages in RCC and normal kidney was slightly lower than T cells (**Figures 8E** **, F**). T cells or NK-T cells did not significantly show this characteristic, and the correlation between them was almost greater than 0.9 (**Figure 8F**). This finding may indicate that T cells have slight difference in gene expression in RCC and normal kidney.

### Integration of scRNA-Seq and GWAS Results and TCGA Data Identified Specific chRCC Cell Type That May Affect Prognosis

All the RCC-related susceptibility gene loci were obtained from the GWAS catalogue (33). After filtering was performed, the susceptible gene loci with *p* value less than 1 × 10^−8^ were selected and matched to corresponding genes (**Table S7**). These susceptible genes were enriched into each cell type of RCC. Twelve of 17 genes associated with RCC were expressed in ccRCC 3 (**Figure S9B**, cluster 15). In type 2 pRCC and chRCC, no aggregation of susceptible genes in one cell type was observed (**Figures S9A** **, C**).

Then, the chRCC data were applied in TCGA to predict the prognosis of three chRCC subpopulations. Six DEGs in chRCC 1 were associated with prognosis (**Figure S9D**), whilst only two were associated with prognosis in chRCC 2 (**Figure S9E**). In addition, 14 of 17 DEGs in chRCC 3 were associated with poor prognosis (**Table S9**). Thus, the composition of different tumor cell types may influence prognosis. chRCC 3 may be a cell type that leads to poor prognosis in chRCC.

### Tumor-Cell Interaction With Ligand–Receptor in CAFs and Immune Cells Discovered the Characteristics of Close Relationships Between Type 2 pRCC and CAFs

**Figure 9:**
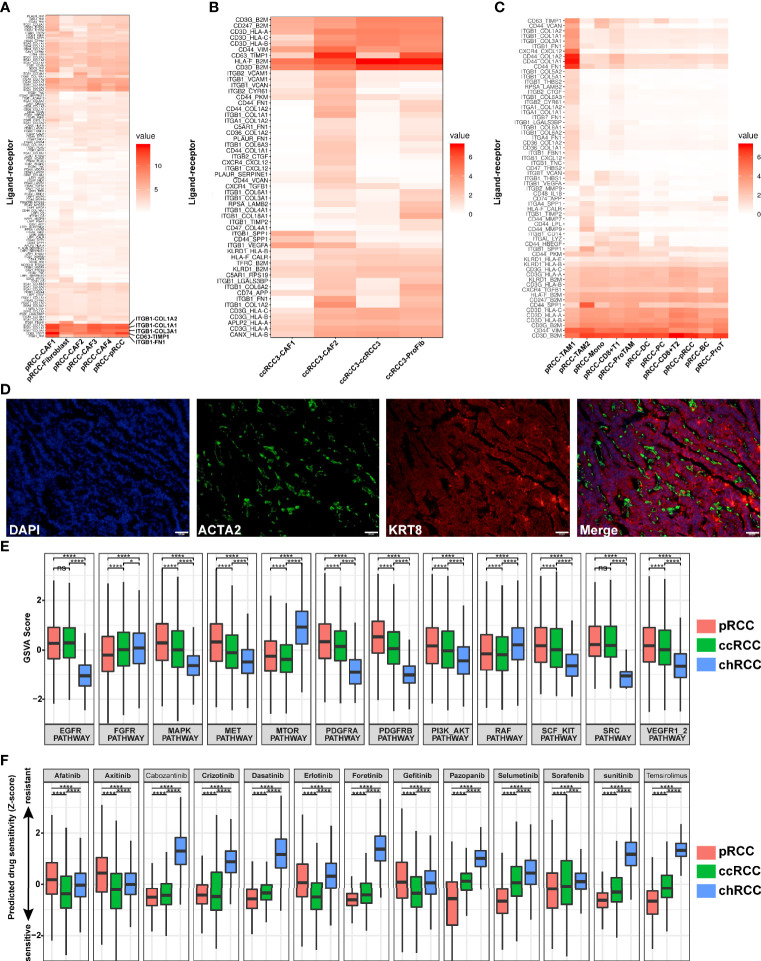
Ligand–receptor interactions in RCC, prediction of drug target pathways and sensitivity to drug responses. **(A)** Ligand–receptor interactions in type 2 pRCC and CAFs. **(B)** Ligand–receptor interactions in ccRCC and CAFs. **(C)** Ligand–receptor interactions in type 2 pRCC and immune cells. **(D)** Spatial CAF location verified in type 2 pRCC using the CAF marker *ACTA2* (green), epithelial cell marker *KRT8* (red) and DNA staining *via* DAPI (blue) within the tissue paraffin sections from human type 2 pRCC samples. Scale bars, 50 μm. **(E)** Prediction of activation of drug target pathways. **(F)** Prediction of activation of drug sensitivity to drug responses. *p < 0.05, ***p < 0.01 and ****p < 0.001.

Here, the ligand–receptor interaction scores between tumor cells and CAFs/immune cells were calculated on the basis of a previous study (36). The cell–cell interaction between tumor cells and CAFs was very close in type 2 pRCC. A total of 118 ligand–receptor interaction scores were greater than 1 (**Figure 9A**). In particular, these ligands–receptors *(ITGB1-COL1A2*, *ITGB1-COL1A1, ITGB1-COL3A1, CD63-TIMP1 and ITGB1-FN1*) interacted more closely between type 2 pRCC and CAFs (**Figure 9A**). CAFs were visualized using immunostaining to understand the spatial location of CAFs within type 2 pRCC. They were located around the tumor cells (**Figure 9D**). The spatial location of CAFs may contribute to their interaction with tumor cells. Interestingly, *ITGB1* upregulation can promote the progression and invasion of gastrointestinal tumors, such as hepatocellular carcinoma and gastric cancer (71, 72). And *ITGB1* Upregulation promotes the development and metastasis of renal cell carcinoma (73). Therefore, the interaction between CAFs and pRCC may promote the progression and invasion of pRCC.

Then, the ligand–receptor interactions between four different ccRCC cell types and CAFs were analysed (**Figures 9B**, **S10A**). CcRCC 3 and CAFs were strongly correlated, especially CAF 2 (**Figure 9B**).Meanwhile, the cell–cell interaction between RCC and immune cells was calculated. In type 2 pRCC, the cell–cell interaction between type 2 pRCC and TAM 1 was strongly correlated (**Figure 9C**). However, the interaction between tumor cells and immune cells was significantly reduced in ccRCC and chRCC (**Figures S10B** **, C**).

### Prediction of Drug Target Pathways and Sensitivity to Drug Responses by scRNA-Seq Results

Given the heterogeneity of tumor cells, the drug target pathways differed amongst various pathologic types of RCC. Using single-cell gene sets involved in drug target pathways and by calculating the GSVA score (37), the relative activation status of the drug target signatures across type 2 pRCC, ccRCC and chRCC was assessed. A total of 12 common drug target pathways were included in this analysis. Most pathways were distinctly regulated in the three pathological types, leading to drug sensitivity difference (**Figure 9E**). Type 2 pRCC and ccRCC were more active than chRCC in RCC classical pathways, such as EGFR and VEGFR pathways, whilst chRCC was more active in MTOR pathway (**Figure 7E**). Subsequently, the sensitivity of 13 targeted drugs in type 2 pRCC, ccRCC and chRCC was predicted. The efficiency of signaling pathway activation for drug sensitivity was predicted on the basis of a ridge regression model (74) and public gene expression profiles and drug sensitivity data were used as a training set (41). After the prediction of drug sensitivity, the targeted drugs (afatinib, axitinib, crizotinib, erlotinib and gefitinib) were more favorably sensitive in ccRCC than in other types of RCC (**Figure 9F**). type 2 pRCC was more sensitive in targeted drugs such as cabozantinib, dasatinib, foretinib, pazopanib, selumetinib, sorafenib, sunitinib and temsirolimus but chRCC was resistant to almost all these 13 targeted drugs (**Figure 9F**).

## Discussion

Bulk RNA sequencing almost reflects the average expression of mRNA in tumor tissues. This expression is difficult to precisely assess only in tumor cells. In the present study, scRNA-seq of multiple pathological types of RCC revealed the transcriptome of tumor tissues at the single-cell level. The tumor cells could be accurately classified into some subpopulations and separated from non-tumor cells (**Figure 2A**). The gene expression characteristics of tumor cells could also be identified (**Figures 2B** **–D**). Tumor-specific markers could be identified by the characteristics of gene expression in tumor cells (**Figures 3A**, **4A, D**). *NDUFA4L2*, considered as a specific marker of ccRCC in a previous study (49), was also highly expressed in type 2 pRCC which was verified by our scRNA-seq and IHC-P results. A previous study reported tumor-specific markers (*RHCG* and *LINC01187*) of chRCC through bulk RNA sequencing data from TCGA (42), similar results and new tumor-specific markers (*SPAG4*) were achieved in the present study using scRNA-seq (**Figures 4E** **, F**). Although RCC a disease with a potentially high level of tumor heterogeneity (13, 75), we have verified that our results are widely feasible through a certain number of samples (**Table S10**). The discovery of these tumor markers may provide a new horizon for the clinical diagnosis of RCC. Therefore, scRNA-seq of tumor is a robust method to discover tumor-specific markers.

In addition, the tumor cell type that may be associated with poor prognosis, such as chRCC 3, could be found. DEG analysis in chRCC 3 showed that most of these DEGs led to poor prognosis (**Table S9**). *SPAG4* was a specific marker of chRCC 3, which showed a tendency to have a poor prognosis but no statistical significance (**Figure S9F**). This result may be reason for the small number of chRCC samples (*n*=64) in TCGA database. A larger sample database is needed to verify this result.

In this study, some subpopulations of CAFs were found in type 2 pRCC and ccRCC (**Figures 6A** **, D**). In type 2 pRCC, most CAFs expressed epithelium markers (*KRT8* and *KRT18*), such as CAF 2, CAF 3 and CAF 4. In previous studies, scholars suggested that EMT is a common source of CAFs (66, 68). In the present study, EMT was hypothesized to be a common biological pattern in type 2 pRCC.

ECs play an important role in tumor growth. Although remarkable progress has been made in the clinical efficacy of anti-vessel drugs, the effect of these agents remains transient (76). Many reasons could be attributed to this result. Therefore, the scRNA-seq of ECs in tumor tissues may provide more valuable biological characteristics. A previous study identified *CLEC14A* as a marker of tumor ECs (77). Indeed, this result was also verified in the present study. In addition, *CLEC14A* was highly expressed in all the captured tumor EC types (**Figures 5B** **, D**). ECs also expressed *VEGFA* or *VEGFC* in type 2 pRCC (**Figure 7B**). Although previous studies have reported how VEGF regulates the growth of ECs (78–80), few studies have reported that tumor ECs self-regulates through autocrine VEGF. Not all tumor ECs possess this characteristic, which may be associated with tumor heterogeneity, and scRNA-seq is a good method to reveal this. Given the samples for scRNA-seq were small, there were some limitation in this study. It is difficult to compare the tumor heterogeneity between more patients who suffered from type 2 pRCC and chRCC. Fortunately, the number of cells captured in each sample was abundant and the transcriptome information of these cells was of high quality. And some of the important results that we discovered with scRNA-seq have been validated by at least 5 different human samples. Thus, we deem that our results are reliable. We hope to apply single-cell multi-omics techniques in future RCC studies, such as chromatin accessibility, cellular transcriptome and spatial transcriptome techniques. The heterogeneity of RCC may be better revealed by integrating multiple dimensions of DNA, mRNA and spatial location at single-cell level.

In conclusion, a comprehensive, single-cell resolution, multiple pathologic transcriptome map of RCC was provided in this study. A number of novel tumor markers of RCC were discovered, which could have a potential value in diagnosing RCC by scRNA-seq. In addition, some new cell types, such as proliferative fibroblast and fibroblast-associated EC, were identified using scRNA-seq. Comparative analysis between normal kidney and RCC enhanced the understanding of tumor-immune microenvironment. Taken together, this study considerably enriched the single-cell transcriptomic information for RCC, which could provide new insights into the diagnosis and treatment of RCC.

## Data Availability Statement

The datasets presented in this study can be found in online repositories. The names of the repository/repositories and accession number(s) can be found below: GEO and accession GSE152938 (https://www.ncbi.nlm.nih.gov/geo/query/acc.cgi?acc=GSE152938).

## Ethics Statement

The studies involving human participants were reviewed and approved by the Institutional Review Board of The First Affiliated Hospital Guangxi Medical University. The patients/participants provided their written informed consent to participate in this study. Written informed consent was obtained from the individual(s) for the publication of any potentially identifiable images or data included in this article.

## Author Contributions

CS performed the scRNA-seq experiments, the IHC and IF experiments and wrote the paper. ZY performed scRNA-seq analyses, created the figures and wrote the paper. YL and WL. performed the scRNA-seq experiments and wrote the paper. YY discussed the draft paper and critically reviewed the manuscript. BG provided assistance during IHC and IF. DL, HY, TL, and QZ provided and dissected RCC and human kidney tissues. YL and WL provided assistance during establishing library. JC and ZM conceived and supervised the project, analysed the data, created the figures, and wrote the paper. All authors contributed to the article and approved the submitted version.

## Funding

This work was supported by the grants from the National Natural Science Foundation of China (81770759), the National Key R&D Program of China (2017YFC0908000), Major Project of Guangxi Innovation Driven (AA18118016), Guangxi key Laboratory for Genomic and Personalized Medicine [grant number 16-380-54, 17-259-45, 19-050-22, 19-185-33, 20-065-33], Guangxi Science and Technology Base and Talent Project (2019AC17009) and Guangxi Clinical Research Center for Urology and Nephrology (2020AC03006).

## Conflict of Interest

The authors declare that the research was conducted in the absence of any commercial or financial relationships that could be construed as a potential conflict of interest.

## Publisher’s Note

All claims expressed in this article are solely those of the authors and do not necessarily represent those of their affiliated organizations, or those of the publisher, the editors and the reviewers. Any product that may be evaluated in this article, or claim that may be made by its manufacturer, is not guaranteed or endorsed by the publisher.
