# Supplementary material for: Single-Cell RNA Sequencing in Multiple Pathologic Types of Renal Cell Carcinoma Revealed Novel Potential Tumor-Specific Markers
Source: Front Oncol. 2021 Oct 14;11:719564. doi: 10.3389/fonc.2021.719564 (PMC8551404; doi:10.3389/fonc.2021.719564)
Supplement: Supplementary file 1 [file DataSheet_1.zip › Supplementary Captions.docx]

Supplementary Material

# Supplementary Tables

**Table S1** Details of single-cell RNA sequencing sample.

**Table S2** Single-cell suspension details for different samples. Quality control (QC).

**Table S3** Differential expression genes (DEGs) of each cluster in type 2 pRCC.

**Table S4** Differential expression genes (DEGs) of each cluster in ccRCC.

**Table S5** Differential expression genes (DEGs) of each cluster in chRCC.

**Table S6** Differential expression genes (DEGs) of each cluster in human kidney.

**Table S7** GWAS identified susceptibility gene associated with RCC.

**Table S8** GO enrichment analysis of biological processes in RCC.

**Table S9** Differential expression genes (DEGs) associated with prognosis in chRCC 3.

**Table S10** Details of IHC-P, IF and Western blot (WB) samples.

# Supplementary Figures

**Figure S1** RCC sample procurement. **(A)** type 2 pRCC **(B)** ccRCC **(C)** ccRCC **(D)** chRCC. The site where the tumour tissue was obtained is highlighted in the picture. The white circles represent tumour sites and the red circles represent normal kidney site.

**Figure S2** Quality control (QC) of the scRNA-seq data. **(A, C)** QC of RCC scRNA-seq data. **(B, D)** QC of normal scRNA-seq data. nFeature, number of genes; nCount, unique molecular identifiers (UMIs); percent.mt, percentage of mitochondrial genes. (C, D) Relationship between the percentage of mitochondrial genes and the mRNA reads, together with the relationship between the amount of mRNA and the reads of mRNA.

**Figure S3** Comparison scRNA-seq data with bulk RNA-seq from TCGA. (**A**) We selected the DEGs (VCAN, NNMT, IGFBP6) of pRCC from TCGA for integration into scRNA-seq results. (**B**) We selected the DEGs (CA9, NDUFA4L2, FABP7) of ccRCC from TCGA for integration into scRNA-seq results. (**C**) We selected the DEGs (KLK1, CFTR, PVALB) of chRCC from TCGA for integration into scRNA-seq results.

**Figure S4** Eliminated batch effects and cell-cycle gene effects. (**A**) UMAP plot representation of 23,951 normal kidney cells from four different samples, including nine distinct cell types. (**B**) Eliminated batch effect between two different ccRCC samples using harmony. (**C**) Eliminated batch effect between four different normal kidney samples using harmony. (**D**) UMAP plot showing the cell cycle status of each cell in type 2 pRCC. (**E**) UMAP plot showing the cell cycle status of each cell in ccRCC. (**F**) UMAP plot showing the cell cycle status of each cell in chRCC. (**G**) UMAP plot showing the cell cycle status of each cell in normal kidney.

**Figure S5** IHC-P performance on the negative control of the normal kidney. **(A–F)** IHC-P verification of the negative control of tumour-specific marker genes *SPOCK1*, *PTGIS*, *NDUFA4L2*, *REG1A*, *RHCG* and *SPAG4*. **(G)** Negative control of IF, staining with PBS as primary antibody.

**Figure S6** Heat map showing the marker genes of each cluster, highlighting the selected marker genes for each cluster. **(A)** Marker genes of type 2 pRCC. **(B)** Marker genes of ccRCC. **(C)** Marker genes of chRCC. **(D)** Marker genes of normal kidney.

**Figure S7** GO enrichment analysis of RCC tumour cells for biological process. **(A)** GO analysis of type 2 pRCC. **(B)** GO analysis of ccRCC1. **(C)** GO analysis of ccRCC2. **(D)** GO analysis of ccRCC3. **(E)** GO analysis of ccRCC4. **(F)** GO analysis of chRCC1. **(G)** GO analysis of chRCC2. **(H)** GO analysis of chRCC3.

**Figure S8** Expression of tumour-specific genes in normal kidney (present scRNA-seq data and previous reported data). **(A, B)** *SPOCK1*, *SLC12A8*, *WISP1*, *PTGIS*, *C5orf46* and *NDUFA4L2* were decreased or not expressed in normal kidney [present data **(A)** and previously reported data **(B)**]. **(C, D)** *REG1A*, *CP* and *FABP7* were also decreased or not expressed in normal kidney [present data **(C)** and previously reported data **(D)**]. **(E, F)** Expression levels of *RHCG*, *LINC01187*, *SPAG4* and *MIR210HG* were decreased in normal kidney [present data **(E)** and previously reported data **(F)**].

**Figure S9** Integrating scRNA-seq data with GWAS results and TCGA data. **(A–C)** Integration of all cell types in three pathological types of RCC and susceptibility genes in RCC as identified by GWAS. X axis, cell clusters; y axis, susceptibility genes. **(A)** pRCC, **(B)** ccRCC and **(C)** chRCC. **(D)** Integration of DEGs from chRCC 1 and TCGA data-identified genes that affected prognosis. **(E)** Integration of DEGs from chRCC 2 and TCGA data-identified genes that affected prognosis. **(F)** Prognosis of *SPAG4* in the TCGA data.

**Figure S10** Quantification of cell–cell interactions occurring in the TME. **(A)** Ligand–receptor interactions in ccRCC and CAFs. **(B)** Ligand–receptor interactions in ccRCC and immune cells. **(C)** Ligand–receptor interactions in chRCC and immune cells (ligand–receptor pairs with interaction score greater than 1 were only shown).
