# Supplementary material for: Single-Cell RNA Sequencing in Multiple Pathologic Types of Renal Cell Carcinoma Revealed Novel Potential Tumor-Specific Markers
Source: Front Oncol. 2021 Oct 14;11:719564. doi: 10.3389/fonc.2021.719564 (PMC8551404; doi:10.3389/fonc.2021.719564)
Supplement: Supplementary file 1 [file DataSheet_1.zip › Supplementary Table 1.DOCX]

**Table S1 Details of single-cell RNA sequencing sample.**

| ID | Hospital | | Age | Sex | Histology | Sample | Sample source |
| --- | --- | --- | --- | --- | --- | --- | --- |
| pRCC | | The First Affiliated Hospital of Guangxi Medical University | 43y | Male | pRCC (type II) | Renal cell carcinoma | This study |
| ccRCC1 | | The First Affiliated Hospital of Guangxi Medical University | 50y | Male | ccRCC | Renal cell carcinoma | This study |
| ccRCC2 | | Affiliated Tumor Hospital of Guangxi Medical University | 41y | Female | ccRCC | Renal cell carcinoma | This study |
| chRCC  and  kidney4 | | The First Affiliated Hospital of Guangxi Medical University | 33y | Male | chRCC | Renal cell carcinoma  and normal kidney | This study |
| kidney1 | | The first affiliated hospital of Guangxi Medical University | 57y | Male | Papillary urothelial carcinoma of the renal pelvis | Normal kidney | GSE131685 |
| kidney2 | | Affiliated tumor hospital of Guangxi Medical University | 59y | Female | ccRCC | Normal kidney | GSE131685 |
| kidney3 | | Affiliated tumor hospital of Guangxi Medical University | 65y | Male | ccRCC | Normal kidney | GSE131685 |

*ccRCC, clear cell renal cell carcinoma; pRCC, papillary renal cell carcinoma; chRCC, chromophobe renal cell carcinoma.
