# Supplementary material for: Single-Cell RNA Sequencing in Multiple Pathologic Types of Renal Cell Carcinoma Revealed Novel Potential Tumor-Specific Markers
Source: Front Oncol. 2021 Oct 14;11:719564. doi: 10.3389/fonc.2021.719564 (PMC8551404; doi:10.3389/fonc.2021.719564)
Supplement: Supplementary file 1 [file DataSheet_1.zip › Supplementary Table 10.DOCX]

**Table S10 Details of IHC-P, IF and Western blot (WB) samples.**

| **ID** | **Age (years)** | **Sex** | **Histology** | **Experiment** |
| --- | --- | --- | --- | --- |
| A1 | 30 | female | pRCC (type II) | IHC-P, IF |
| A2 | 40 | female | pRCC (type II) | IHC-P, IF |
| A3 | 59 | male | pRCC (type II) | IHC-P, IF |
| A4 | 46 | male | pRCC (type II) | IHC-P, IF |
| A5 | 44 | male | pRCC (type II) | IHC-P |
| A6 | 54 | female | ccRCC | IHC-P, IF |
| A7 | 66 | female | ccRCC | IHC-P |
| A8 | 60 | male | ccRCC | IHC-P, IF |
| A9 | 51 | male | ccRCC | IHC-P, IF |
| A10 | 61 | female | ccRCC | IHC-P, IF |
| A11 | 43 | female | chRCC | IHC-P |
| A12 | 62 | female | chRCC | IHC-P |
| A13 | 50 | male | chRCC | IHC-P |
| A14 | 65 | female | chRCC | IHC-P |
| A15 | 34 | male | chRCC | IHC-P |
| RN1 | 43 | male | Normal kidney | IHC-P |
| RN2 | 50 | male | Normal kidney | IHC-P |
| RN3 | 67 | male | Normal kidney | IHC-P |
| RN4 | 56 | female | Normal kidney | IHC-P |
| RN5 | 41 | female | Normal kidney | IHC-P |
| RN6 | 49 | female | Normal kidney | IHC-P |
| RN7 | 33 | male | Normal kidney | IHC-P |
| RN8 | 41 | male | Normal kidney | IHC-P |
| ccRCC28 | 63 | male | ccRCC and normal kidney | WB |
| ccRCC47 | 57 | male | ccRCC and normal kidney | WB |
| ccRCC48 | 45 | male | ccRCC and normal kidney | WB |
| ccRCC50 | 64 | male | ccRCC and normal kidney | WB |
| ccRCC52 | 27 | male | ccRCC and normal kidney | WB |
