# Supplementary material for: Single-Cell RNA Sequencing in Multiple Pathologic Types of Renal Cell Carcinoma Revealed Novel Potential Tumor-Specific Markers
Source: Front Oncol. 2021 Oct 14;11:719564. doi: 10.3389/fonc.2021.719564 (PMC8551404; doi:10.3389/fonc.2021.719564)
Supplement: Supplementary file 1 [file DataSheet_1.zip › Supplementary Table 2.DOCX]

**Table S2 Single-cell suspension details for different samples. Quality control (QC).**

| **Sample** | **Viability (%)** | **Capture cells** | **Cells after QC** |
| --- | --- | --- | --- |
| pRCC | 87.25 | 11949 | 10132 |
| ccRCC1 | 86.4 | 10762 | 5665 |
| ccRCC2 | 91.8 | 9837 | 7250 |
| chRCC  kidney1  kidney2  kidney3 | 91.1  94.4  98  90 | 10254  8091  6472  10705 | 7216  7221  5543  10602 |
| kidney4 | 87.8 | 4800 | 585 |
