# Supplementary material for: Single-Cell RNA Sequencing in Multiple Pathologic Types of Renal Cell Carcinoma Revealed Novel Potential Tumor-Specific Markers
Source: Front Oncol. 2021 Oct 14;11:719564. doi: 10.3389/fonc.2021.719564 (PMC8551404; doi:10.3389/fonc.2021.719564)
Supplement: Supplementary file 1 [file DataSheet_1.zip › Supplementary Table 9.DOCX]

**Table S9 Differential expression genes (DEGs) associated with prognosis in chRCC 3.**

| **Gene** | ***p* (Logrank)** | **prognosis** |
| --- | --- | --- |
| *ACTG1* | 0.049 | poor |
| *BNIP3* | 0.00039 | poor |
| *C4orf3* | 0.019 | poor |
| *DDIT4* | 0.048 | poor |
| *ENO1* | 0.0076 | poor |
| *GAPDH* | 0.0032 | poor |
| *HILPDA* | 0.00091 | poor |
| *LDHA* | 0.0016 | poor |
| *MIR210HG* | 0.014 | poor |
| *NPM1* | 0.019 | poor |
| *P4HA1* | 0.0034 | poor |
| *RPL32* | 0.045 | poor |
| *RPS13* | 0.0019 | poor |
| *TPI1* | 0.012 | poor |
| *CLDN3* | 0.00099 | favorable |
| *CRYAB* | 0.012 | favorable |
| *IER2* | 0.011 | favorable |
